# Theoretical Investigation of the Nonlinear General Rate Model with the Bi-Langmuir Adsorption Isotherm Using Core–Shell Adsorbents

**DOI:** 10.1021/acsomega.3c06023

**Published:** 2023-11-10

**Authors:** Muhammad Afraz Rasheed, Sadia Perveen, Shamsul Qamar

**Affiliations:** † Department of Mathematics, Air University, PAF Complex, Sector E-9, Islamabad 44230, Pakistan; ‡ Department of Mathematics, COMSATS University, Park Road, Islamabad 45550, Pakistan

## Abstract

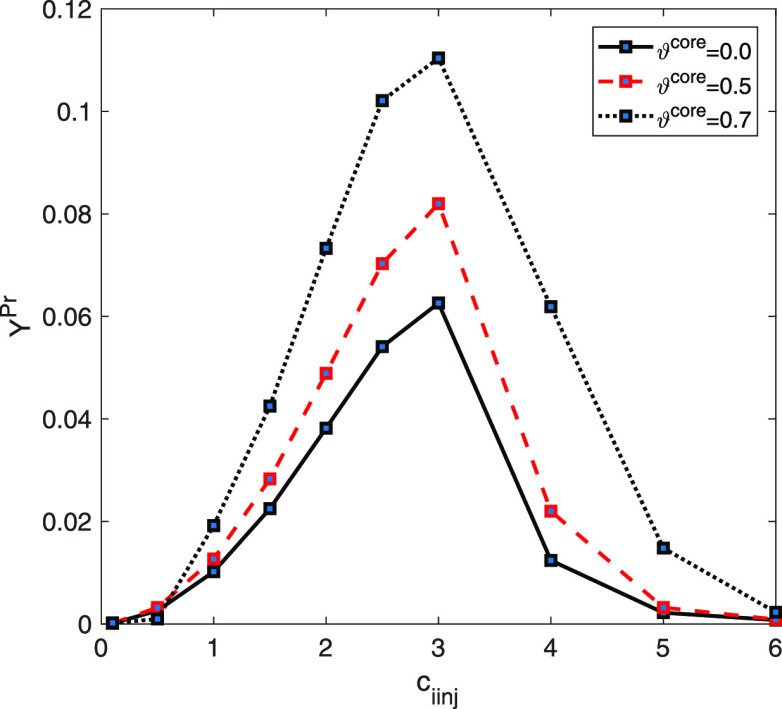

Core–shell particles enable the separation of intricate mixtures in a highly efficient and rapid manner. The porous shell particles increased the separation efficiency with expedited flow rates due to an abatement in the pore volume accessible for longitudinal diffusion and a decrease in diffusion path length. This study focuses on the numerical approximation of a nonlinear isothermal general rate model applied to stationary bed columns that are replete with inert core adsorbents featuring double adsorption sites. The transport of solute in heterogeneous porous media can be modeled by a nonlinear convection acquiescent partial differential equation system together with a specific nonlinear algebraic relation: the bi-Langmuir adsorption isotherm. Therefore, it is important to develop accurate and reliable numerical techniques that can perform accurate numerical simulations of these models. We extended and implemented a second-order, semidiscrete, high-resolution finite volume method to simulate the governing equations of the model. Single solute flow and multi component mixture flows are assessed through a series of numerical experiments to theoretically illustrate the repercussions of intraparticle diffusion, film mass resistance, axial dispersion, and the size of the inert core radius upon simulated elution curves. Standard performance criteria are assessed to determine the optimal core radius fraction range for optimizing the separation performance.

## 1 Introduction

High-performance liquid chromatography, also known as HPLC, is widely recognized as an indispensable analytical instrument used in numerous disciplines, including manufacturing, pharmaceuticals, biotechnology, environmental analysis, clinical testing, and diagnostics. Presently, the emphasis in HPLC is centered on achieving a reduced analysis time, enhanced efficiency of kinetic processes, and decreased back pressure when working with a wide range of sample types. To enhance the capabilities of HPLC, ongoing efforts have been made to strengthen and reduce the particle size of the particles packed within the columns. The objective of these advancements is to boost the overall effectiveness and performance of the HPLC systems. The emergence of ultra HPLC (UHPLC) incorporating small particles has led to advancements in the analytical capabilities of HPLC and improved column performance.^1^

Solid core particles have revolutionized UHPLC by offering enhanced efficiency and faster separations compared with traditional fully porous particles. These solid core particles, identified as extraneously porous particles or shell particles, possess a solid silica core with a thin porous shell coating.^2^ This unique structure combines the advantages of both fully porous particles and fully solid particles, leading to improved chromatographic performance.^3^ The use of solid core particles in the UHPLC provides several benefits. First, their smaller particle size results in reduced diffusion paths and improved mass transfer, enabling faster and more efficient separations. This leads to shorter analysis times, increased sample throughput, and improved productivity in various analytical applications.^4,5^ The use of solid core particles in UHPLC also addresses the challenge of high back pressure commonly associated with smaller particle sizes, typically ranging from 1.7 to 2.7 μm. Due to their distinctive structure, solid core particles offer reduced solvent consumption, making them an environmentally friendly option. They also offer reduced flow resistance, enabling high-speed separations with minimal effect on system back pressure.^6^ This permits the use of speedy flow rates, thereby reducing analysis time further without compromising separation quality.^7–9^ Another advantage of solid core particles is their compatibility with conventional HPLC instruments. They can be used with existing UHPLC systems without requiring significant modifications or investments in new equipment. This makes the transition from traditional HPLC to UHPLC with solid core particles more accessible and cost-effective.^10,11^

The published literature encompasses various studies that highlight the utilization of particles with a core–shell structure for quantifying an extensive range of analytes in different matrices. These studies involve employing diverse pretreatment methods and a variety of detectors to achieve accurate results. Reversed-phase HPLC predominantly employs columns filled with core–shell particles, where they exhibit a significant reduction in plate height, commonly up to 1.7 times.^12,13^ A comparison of analytical performance between narrow bore core–shell columns, totally porous columns, and monolithic columns has demonstrated superior results in terms of both efficiency and peak asymmetry factors.^14,15^ The results reported in ref (16) for chiral separation using core–shell particles were comparable to those achieved with completely porous particles. The utilization of these groundbreaking columns offers opportunities for improving analysis in the field of comprehensive 2D liquid chromatography. They address the demand for rapid separations in the second dimension despite the associated high operational pressure that arises when using columns densely packed with sub-2 μm particles, as discussed in the comprehensive review by Jandera et al. in ref (17)

In the realm of HPLC, the utilization of solid core particles strikes a balance among completely porous particles and nonporous particles, as elaborated in the study by Wang.^18^ Nonporous particles effectively prevent intra particle diffusion and yield sharp elution peaks with minimal retention times, as evidenced by the findings presented in the studies by Lee, Rissler, Xiang, Fekete, and Gu.^19–23^ On the contrary, fully porous particles exhibit the largest differences in retention time but are prone to excessive broadening of the chromatographic band. Comparative experiments and analyses of various commercially accessible completely porous and cored beads were conducted^6,24^ to assess their performance and characteristics.

The utilization of mechanistic-based models not only facilitates process development and optimization but also enables enhanced process control and automation. The objective of mechanistic modeling is to depict mathematical expressions that capture the physical and chemical interactions among the involved components. Mathematical modeling of chromatographic processes holds significant importance in advancing chromatographic science by providing (a) insights into separation mechanisms, (b) optimizing chromatographic conditions, (c) predicting behavior, (d) aiding scale up, and (e) optimizing cost and time efficiency.^25^ In liquid chromatography, the theory of chromatography has provided a vast array of mass transport models for solute molecules.^26–28^ These models can be categorized into three main classes: equilibrium theory, plate theory, and rate models. Among the available mathematical models, the general rate model (GRM) in one dimension stands out as one of the most extensively employed ones. The lumped kinetic model (LKM) and equilibrium dispersive model (EDM) are also commonly used, providing a simplified approach to modeling. The detailed expression of specific molecular interactions between phases is achieved by incorporating various binding models. These binding models can take the form of kinetic equations, lumped mass-transfer kinetic equations, or adsorption isotherms.^27,29,30^

To obtain intraparticle diffusion coefficients, a GRM is applied to core beads in the study by Zhou.^4^ The study conducted by Kaczmarski and Guiochon involved an examination of coated beads with narrow shells by employing a lumped particle model. They suspected that a single average concentration value could represent the breakthrough profile of concentration within the thin shell.^31^ To establish a comparison with completely porous particles, they employed a GRM specifically designed for such particles. The optimization of core size for linear chromatography, with the aim of minimizing the height equivalent theoretical plate (HETP) number, was performed by Li in ref (32). Furthermore, the investigation conducted by the researchers explored the realm of protein adsorption within the process of expanded bed adsorption. They accomplished this by employing the GRM in conjunction with the Langmuir isotherm, as examined in studies by Li.^33,34^ Additionally, they derived an analytical solution for the linear GRM as described in refs (35) and (36), to anticipate the breakthrough plots for the inert core-based adsorbent. The articles by Gu and Qamar^23,37^ discuss a nonlinear GRM for core beads and elaborate on its numerical solution strategy. In a separate study, Luo et al.^38^ employed the GRM to simulate cored particles in size-exclusion chromatography.

The main focus of this paper revolves around the numerical approximation of a nonlinear GRM applied to inert solid core particles. In particular, the paper concentrates on core–shell particles that exhibit heterogeneity in the adsorbent surface, distinguished by the bi-Langmuir adsorption isotherm.^39–42^ The paramount innovation presented in this paper lies in the extensive array of features encapsulated within the one-dimensional model for nonlinear chromatography, elucidated through the acquired numerical solutions. The nonlinear general rate model (GRM) comprises a set of partial differential equations (PDEs) for which a closed-form solution does not exist. Consequently, numerical methods are employed to calculate approximate solutions. Mass transport problems involving convection, such as the GRM, are susceptible to shocks that can emerge due to steep gradients in the solutions. The literature encompasses various methods for solving conservation law PDEs through discretization techniques like finite difference (FD),^43^ finite volume (FV),^44^ finite element (FE),^45^ and discontinuous Galerkin (DG).^46^ Stabilization techniques, such as weighted essentially nonoscillatory (WENO)^47^ or total-variation-diminishing (TVD),^48^ are occasionally combined with discretization methods. The strategy for numerically solving the current complex nonlinear model equations relies on the utilization of a precise and efficient high-resolution finite volume method (HR-FVM). Some practical case studies involving the analysis of single solute flow, two component mixtures, and three-component mixtures of relevance are conducted. The aim of these case studies is to examine the impact of fractional core radius, axial dispersion, resistance to film mass transfer, and resistance to intraparticle diffusion on the elution curves. To comprehend the procedure and enhance the core size for attaining optimum productivity, assessment criteria are introduced. A contrast among linear, Langmuir, and bi-Langmuir cases is addressed to elucidate the influence of nonlinearity. Understanding and characterizing nonlinear adsorption conditions are crucial for optimizing chromatographic separations. It allows for the selection of appropriate stationary phases, column dimensions, and operating conditions to achieve the desired separation outcomes. The developed tools and the generated simulation results should be beneficial in synthesizing custom-made particles.

The organization of this paper is structured in the following manner: Section 2 presents the GRM for core–shell particles, considering bi-Langmuir adsorption. Section 3 presents a discussion of the numerical solution procedure utilizing the HR-FVM. Section 4 introduces the process specification criteria used for evaluating the implementation of preparative chromatography. Section 5 presents numerical simulations along with a discussion of the obtained results. In Section 6, concluding remarks are compiled.

## 2 Nonlinear GRM for Core–Shell Particles

For modeling multicomponent LC, adsorption in an isothermal column filled with inert solid core particles having two adsorption sites is considered. The GRM describes the convective transport of solute molecules through the column interstitial volume, the broadening of the band due to dispersion along the axial axis of the moving mobile phase, the mass transfer resistance through a stagnant film surrounding the beads, diffusion via the pores of porous particles, and adsorption onto the inner surfaces of microscopic beads. Thus, multicomponent GRM for LC is composed of (i) a mass-transfer PDE for the bulk fluid phase and (ii) a mass-transfer PDE for particle phase was coupled by an equation (adsorption isotherm) to describe the eluite-stationary binding mechanism. The model equations for mass balance in the column bulk-fluid phase and the stationary liquid phase within particle macro-pores are illustrated based on continuity equations deduced from the theory of conventional transport phenomena.^49^
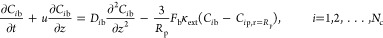
1
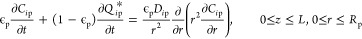
2

**Figure 1:**
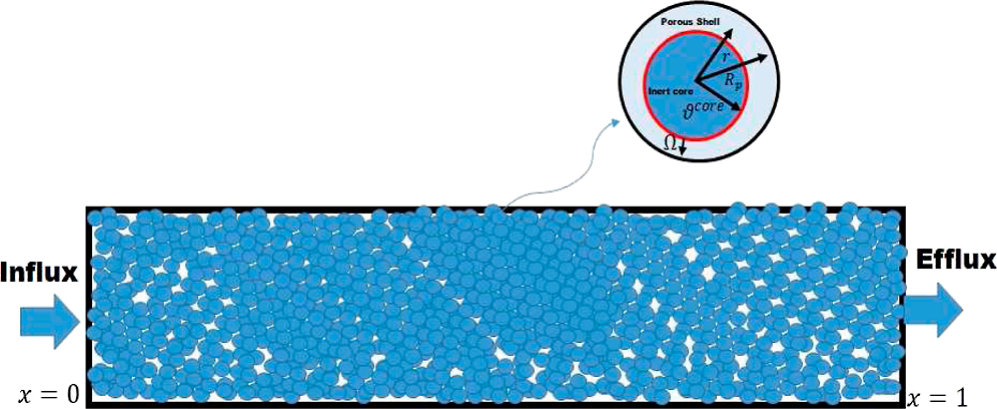
Diagrammatic illustration of a stationary bed adsorber along with a spherical cored particles comprising a porous shell and a nonporous solid core.

The assumption is made that the particle size, denoted as *R*_p_, and the core size, denoted as *R*^core^, of cored particles are uniform. While the inner core is impenetrable, the outer shell is permeable to diffusion while preventing convection. In the above equations, *C*_*i*b_ represents the concentration of the eluite species *i* in the bulk fluid, while *C*_*i*p_ represents the concentration of the same species in the pores of the particle. The phase ratio, denoted as *F*_b_, is defined as (1 – ϵ_b_)/ϵ_b_, where ϵ_b_ represents the void fraction of the stationary bed, *u* represents the superficial velocity of the mobile phase, *D*_*i*b_ characterizes the extent of axial dispersion in the bulk fluid, and κ_ext_ represents the coefficient associated with mass transfer at the external film interface. The variables *t* and *z* represent the temporal and axial coordinates of the column, respectively. Furthermore, within the beads, the radial coordinate is denoted as *r*, the precise concentration of the mixture in the stationary phase shell is represented as *Q*_*i*p_^*^, ϵ_p_ represents the particle’s internal porosity, and *D*_*i*p_ represents the macro pore diffusivity of species *i* (c.f. Figure 1).

The bi-Langmuir adsorption isotherm equation, which characterizes the competitive retention behavior exhibited by multicompound mixtures, is
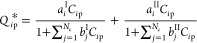
3where, *a*_*i*_^I^, *a*_*i*_^II^, *b*_*i*_^I^, and *b*_*i*_^II^ represent the equilibrium Henry constants and the nonlinearity extent constants for the nonselective and selective binding sites I and II, respectively, of the immobile solid phase. The implementation of dimensionless equations of mass balance facilitates the evaluation of the effect of particular kinetic parameters in a substantial manner. The aforementioned PDE system is nondimensionalized by employing the dimensionless variables and parameters listed below in order to reduce the number of variables.
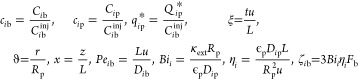
4In eq 4, *C*_*i*b_^inj^ stands for the *i*th species nonzero injected bulk concentration, *Pe*_*i*b_ is the column-length-based Peclet number, *Bi*_*i*_ is the specific Biot number, and the symbol η_*i*_ defines the *i*th compound’s space-time to intra-particle diffusion time ratio. The coupled PDE system given in eqs 1–3 can be rewritten by incorporating the above-mentioned scaled parameters as
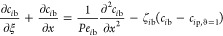
5
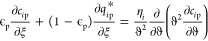
6
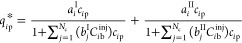
7Equations 5 and 6 are linked at a radius *r* = *R*_p_ by means of a subsequent expression that measures the time-dependent variation of the mean adsorption capacity of the particles
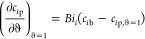
8

Moreover, *q*_*i*p_^*^ in eq 7 is the dimensionless upper concentration in the solid phase. *q*_*i*p_^*^ can be determined by quantifying the concentration of eluites in the fluid contained within the particle macropores *c*_*i*p_. The range of ϑ-axis in eq 6 is from 0 to 1 for completely porous particles. While for cored particles, it ranges from ϑ^core^ = *R*^core^/*R*_p_. Variable core radius is essential, as this investigation focuses on cored particles with any fraction of cored radius ϑ^core^. For cored particles, ϑ^core^ ≠ 0, i.e, ϑ^core^ ≤ ϑ ≤ 1 (c.f. eq 6), whereas for completely porous particles, ϑ^core^ = 0. In accordance with ref (23), it is preferable to supplant the ϑ-axis by 0 ≤ Ω ≤ 1, where
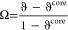
9By replacing
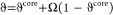
10in eqs 5 and 6 and utilizing eq 7, they produce
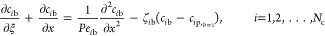
11
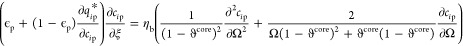
12

The GRM of the inert core adsorbent presented in eqs 11 and 12 requires the following initial and boundary conditions to be provided. The initial conditions of a regenerated column are described as follows
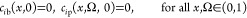
13For eq 12 (c.f. eq 8) at Ω = 0 and Ω = 1, the following boundary conditions were considered.
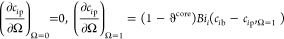
14Equation 11 requires adequate inflow and outflow boundary conditions (BCs). In the present scenario, the column inlet is subjected to the Robin-type boundary condition, also referred to as Danckwerts boundary conditions in the field of chemical engineering (c.f.^50^)
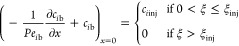
15where, *c*_*i*inj_ represents the dimensionless constant feed concentration for a given dimensionless injection time period ξ_*inj*_, respectively. A zero gradient of fluid concentration modeled by Neumann outflow boundary conditions is employed at the exit of the chromatographic column ı.e at *x* = 1
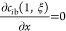
16

## 3 Derivation of Finite Volume Scheme

Numerical simulation of the GRM with the multicomponent bi-Langmuir adsorption isotherm for adsorption liquid chromatography is challenging due to the nonlinear coupling between the state variable and the emergence of precipitous concentration fronts. In this section, a high-resolution semidiscrete finite volume method is extended and derived to solve the PDE system incorporating core–shell particles in the model equations.^51,52^ Steps in the generic numerical solution technique include: (i) discretization of spatial derivatives transforms the PDE system into time-dependent ODEs and (ii) numerical integration methods solve ODEs by discretizing into a nonlinear algebraic equations (AEs) system and solving iteratively.

Taking into account the bi-Langmuir isotherm (c.f eq 7) for a sample mixture consisting of three constituents, we derive the subsequent compact system of PDEs from model eqs 11 and 12
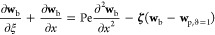
17
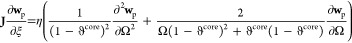
18where
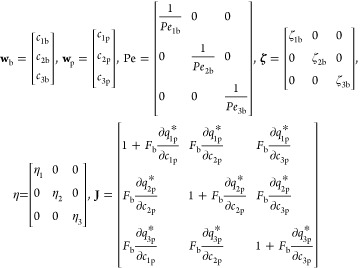
19Equations 18 and 19 represent a system of nonlinear PDEs. The spatial domain of these equations is first discretized by using a finite volume scheme. Domain discretization yields a nonlinear ODE system.

### 3.1 Domain Discretization

Considering that  and  represent the total number of nodes in *x* and Ω-coordinate, respectively. Considering a Cartesian mesh with a computational domain of [0, 1] × [0, 1]. Let the mesh cells for the computational domain of the specified problem be represented by Ψ_*uv*_ = [*x*_*u*–1/2_, *x*_*u*+1/2_] × [Ω_*v*–1/2_,Ω_*v*+1/2_] for  and . The initial vertex and the individual nodes (*x*_*u*_,Ω_*v*_) in the cell Ψ_*uv*_ are defined as
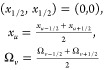
20and the constant step size for the mesh with uniform spacing is
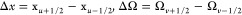
21Because **w**_b_ = *c*_*i*b_(*x*,ξ) and **w**_p_ = *c*_*i*p_(*x*,Ω,ξ), therefore, for *I*_*u*_ = [x_*u*–1/2_,x_*u*+1/2_] and Ψ_*uv*_ = [*x*_*u*–1/2_,x_*u*+1/2_] × [Ω_*v*–1/2_,Ω_*v*+1/2_], the averaged values *c*_b,*u*_(ξ) and *c*_p,*u*,*v*_(ξ) of the cell Ψ_*uv*_ at any time ξ are interpreted as
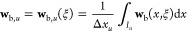
22
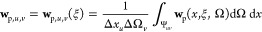
23Integrating eq 17 over the interval *I*_*u*_ and using eqs 22 and 23, we derive
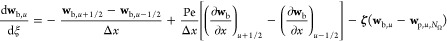
24where *u* = 1, 2, ..., *N*_*x*_, approximating the differential term of the axial diffusion component as
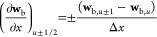
25Integration of eq 18 over the interval Ψ_*uv*_ yields
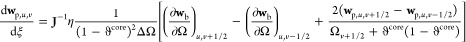
26where
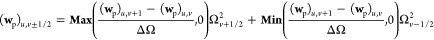
27In addition, for eqs 24 and 26, approximations of concentration values are needed at the cell interfaces x_*u*±1/2_and Ω_*v*±1/2_. There are numerous methods for approximating these fluxes, resulting in an assortment of numerical schemes. Here, we present the first- and second-order methods, accompanied by the TVD-RK scheme, employed to attain a second-order level of temporal accuracy.

### 3.2 First-Order Cell Interface Concentration Approximation

Because all vector components **Pe** and  are non-negative, the vectors of concentration **w**_b_ and **w**_p_ at the cell interfaces are approximated using a backward difference formula as
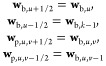
28The preceding approximations provide first-order accuracy for the scheme’s axial and particle radial coordinates.

### 3.3 Second-Order Cell Interface Concentration Approximation

The concentrations at the cell interface are evaluated as follows to obtain a second-order accurate scheme
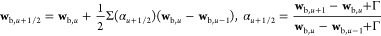
29
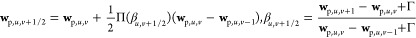
30The eqs 29 and 30 generate a high-resolution flux limiting scheme. In this case, the value of Γ = 10^–10^ is employed to prevent division by zero. The numerical method’s local monotonicity (positivity), as indicated in refs^44^, (52), and (53) is preserved through the utilization of flux limiting functions Σ and Π, ensuring the maintenance of desired properties. They are characterized as
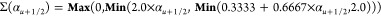
31
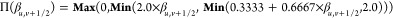
32

The high-resolution scheme suggested by eqs 29 and 30is not suitable for the boundary interval. Accordingly, the boundary intervals are approximated by using first-order backward approximations. eqs 29 and 30 are used to determine the fluxes at each subsequent interior interval. It should be emphasized that the utilization of a first-order backward scheme at the boundary cells does not compromise the overall accuracy of this method.

### 3.4 ODE-Solver

In order to guarantee the same level of second-order precision in the time coordinate, a second-order accurate TVD-RK method is utilized to find a solution for eqs 29–32.^52^ Denoting the right-hand-side of eqs 29 and 30 by ϒ(**w**_b_,**w**_p_,_Ω=1_) and χ(**c**_p_), a second-order TVD RK scheme updates **w**_b_ and **w**_p_, by means of the subsequent two stages
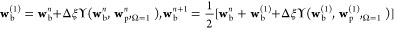
33
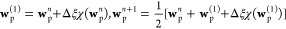
34In the given context, **w**_b_^*n*^ and **w**_p_^*n*^ represent the solutions obtained at the previous time step, denoted by ξ^*n*^. On the other hand, **w**_b_^*n*+1^ and **w**_p_^*n*+1^ correspond to the updated solutions at the next time step, referred to as ξ^*n*+1^. Furthermore, the time step, denoted by Δξ, is determined based on the Courant–Friedrichs–Lewy (CFL) conditions
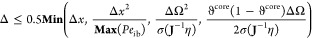
35

In the given context, the symbol σ denotes the spectral radius of a matrix. The aforementioned numerical algorithm was implemented by using the C programming language, employing a grid with dimensions of 100 × 80 points. The program was executed on a laptop computer equipped with a 12th Gen Intel(R) Core(TM) i7-1255U processor running at a clock speed of 1.70 GHz. The computer also had a random access memory (RAM) capacity of 40 GB.

## 4 Process Specifications for Performance

In industrial applications, the optimization of preparative chromatographic processes is crucial in terms of enhancing the efficiency, productivity, and yield. Hovath and Fellinger^3^ have introduced a criterion for evaluating the performance of chromatographic separations and improving product quality. This criterion can be employed to improve the overall quality of the separated compound through a systematic approach to optimizing critical parameters. To formulate this benchmark precedent, we examine a two component mixture where the reference affinity of component two for sites I and II of the stationary phase is higher than that of component one. Specifically, we assume that *a*_1_^I^ < *a*_2_^I^ and *a*_1_^II^ < *a*_2_^II^. Assuming that ξ^1^ represents the nondimensional time at which the fraction of component one exceeds a specific threshold (i.e., ), where . Similarly, ξ^2^ corresponds to a nondimensional time when the concentration of component two decreases below a specific level .

### 4.1 Cycle Time

The time duration between two consecutive injections is referred to as the cycle time, which can be defined as
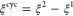
36

### 4.2 Purity

The point at which the fractionation of component one ceases is commonly known as the cut time. In our calculations, we employ the following expression to ascertain the cut time, denoted as ξ^cut^ for component one
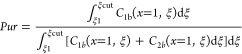
37

The designated purity level, determined by the peak area, was established at 99%.

### 4.3 Productivity

The term “reduced productivity” *Y*^Pr^ represents the desired quantity of the compound produced within a specific time cycle. For component one, It is evaluated as
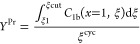
38

The conversion of this reduced productivity into its conventional dimensional form can be accomplished simply by scaling it up with the volumetric flow rate.

### 4.4 Yield

The recovery yield is determined by the ratio of the quantity of the required constituent in the purified fraction to the quantity initially injected at the column entrance. For the first eluting component, the recovery yield is defined as
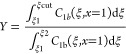
39

## 5 Results and Discussion

Within this section, some selected numerical case studies are presented to examine the impact of ϑ^core^, *Pe*_*i*b_, η_*i*_, and *Bi*_*i*_ on the break through curves. Moreover, the impacts of ϑ^core^, *c*_*i*inj_, *Bi*_*i*_, and η_*i*_ on productivity and recovery yield are analyzed. The case studies include elution with a single component (*N*_c_ = 1), two components (*N*_c_ = 2), and three components (*N*_c_ = 3). The dimensionless injection time for all the components in the simulated results is ξ_inj_ = 1.0. All representative simulation parameters of the numerical test problems are given in Table 1.

**Table 1:** Compilation of Model-Parameters Utilized in the Simulation Studies

figure Nr	Comp. Nr	*Pe*_*i*b_	*Bi*_*i*_	η_*i*_	ϵ_b_	ϵ_p_	*C*_*i*b_^inj^	*a*_*i*_^I^	*a*_*i*_^II^	*b*_*i*_^I^	*b*_*i*_^II^
2 & 3	1	1500	50	2.0	0.4	0.5	1.0	10	15	0.5	1.0
4	1										
	2	1500	50	2.0	0.4	0.5	1.0	30	35	1.5	2.0
5	1										
	2										
6	1						[0.1–7]				
	2						[0.1–7]				
7	1						3.0				
	2						3.0				
8	1		5, 50, 150				1.0				
	2		5, 50, 150				1.0				
9	1			0.5, 2, 2.5			1.0				
	2			0.5, 2, 2.5			1.0				
10	1										
	2										
	3	1500	50	2.0	0.4	0.5	1.0	70	75	3.0	3.5

### 5.1 Elution of a Single Solute Flow

**Figure 2:**
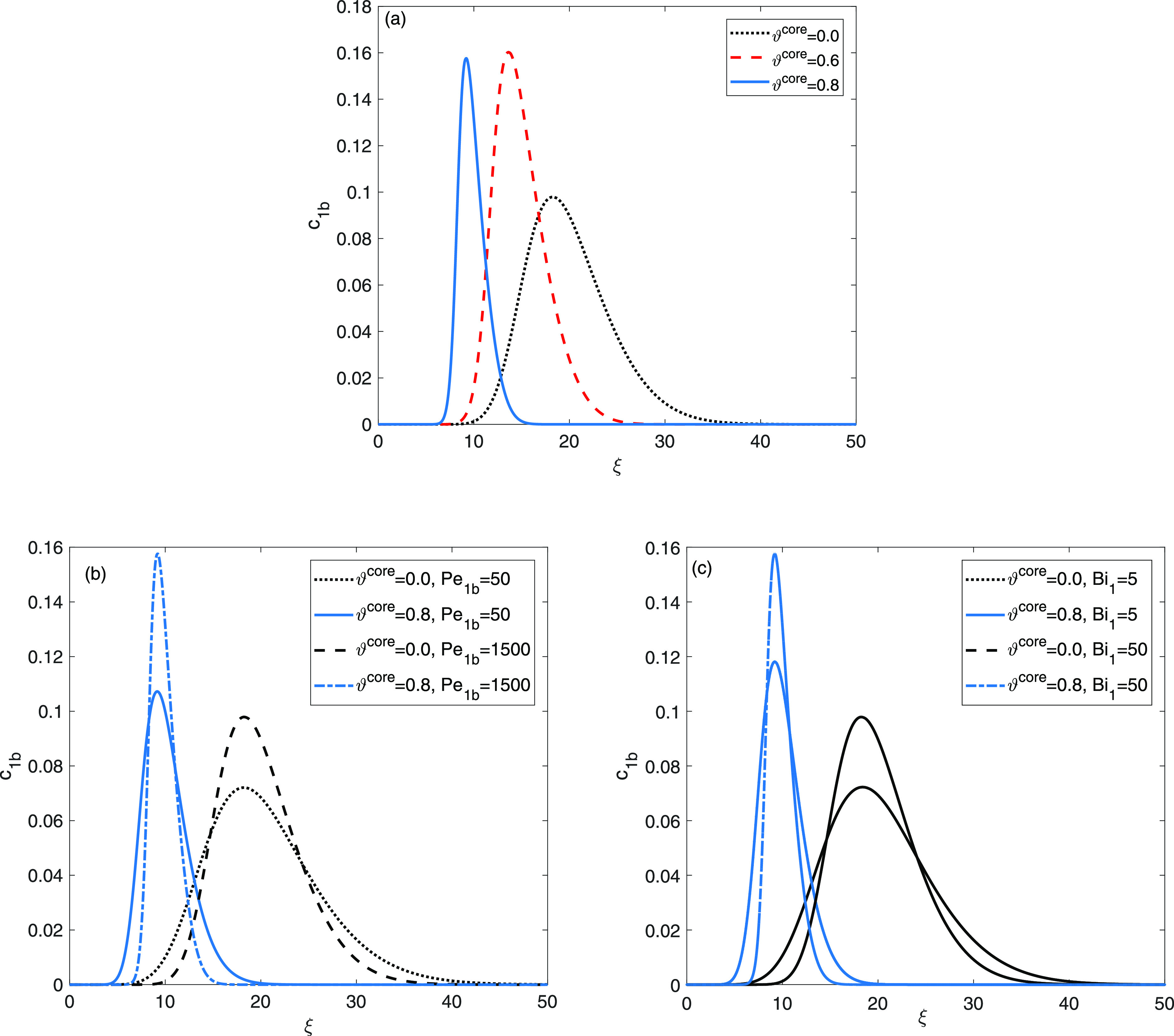
Single solute flow: investigation into the impacts of (a) core radius fraction ϑ^core^, (b) Peclet number *Pe*_1b_, and (c) Biot number *Bi*_1_ on the elution curves.

Figure 2a demonstrates a collation of the breakthrough profiles for various core radius fractions and entirely porous particles for an elution of a single solute flow system. By augmenting the fraction of the core radius ϑ^core^ form 0 to 0.8, it can be observed that the elution profiles become more distinct, resulting in sharper peak profiles with shorter retention times.

This indicates an enhancement in the column’s efficiency and a decrease in its capacity when transitioning from completely porous particles to particles with a thin shell. The sharpening of the peaks is attributed to a reduction in the resistance encountered during mass transfer within pores. The shorter residence times can be attributed to the loss of nonuniform binding sites on the surface of stationary phase as the ϑ^core^ value increases. The exploitation of core–shell particles results in a reduction in the adsorption strength of the column, and a decline in the thickness of the porous shell may lead to a diminishing separation capacity of the column. Figure 2b,c illustrates the impact of model-parameters *Pe*_1b_ and *Bi*_1_ on the effluent profiles for two distinct values of ϑ^core^. If there is significant axial dispersion or resistance to mass transfer within the film, it becomes evident that the peaks widen and the corresponding times for peak maxima are inappreciably cut down. The influence of ϑ^core^ is consistent across all plots.

**Figure 3:**
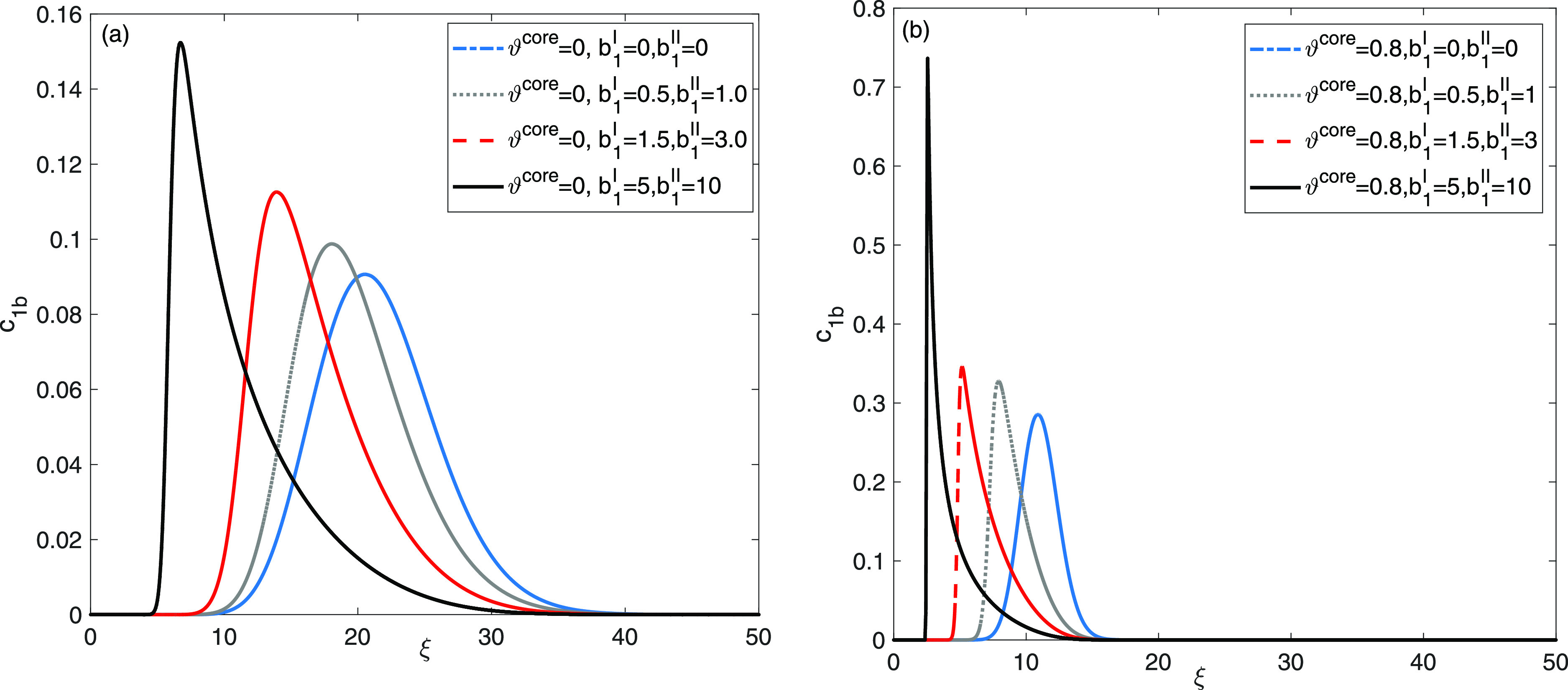
Single solute flow: investigation into the impacts of extent of nonlinearity coefficients for site I and site II, (a): for ϑ^core^ = 0 and (b): ϑ^core^ = 0.8.

Figure 3 examines the elution curves of a single solute flow to investigate the intricacies of the entire adsorption–desorption process, which is influenced by both intraparticle diffusion and the presence of nonuniform sites for adsorption on the surface of the stationary phase. The presence of double adsorption sites led to diverse behaviors of the solutes within the column, consequently impacting peak tailing. The elution profile are generated by assuming ϑ^core^ = 0.0, ϑ^core^ = 0.8. Moreover, four different sets of values for the extent of nonlinearity coefficient are considered, (a). *b*_1_^I^ = 0 and *b*_1_^II^ = 0 (b). *b*_1_^I^ = = 0.5 and *b*_1_^II^ = 1.0, (c). *b*_1_^I^ = = 1.5 and *b*_1_^II^ = 3.0, and (d). *b*_1_^I^ = 5, *b*_1_^II^ = 10, respectively. The simulation result shows a transition from a Gaussian shape, indicating linear behavior (*b*_1_^I^ = 0, *b*_1_^II^ = 0), to asymmetrical shaped profiles, indicating nonlinear behavior, when (*b*_1_^I^ = 5, *b*_1_^II^ = 10). Higher values of adsorption energy coefficients reveal asymmetric and self-sharpening patterns in breakthrough profiles, which indicate variations in the adsorption activity of the eluent on two distinct nonuniform sites for adsorption. Notably, a significant increase in peak heights and a reduction in retention times of the elution profiles are clearly observed when the adsorption energy coefficients are set to *b*_1_^I^ = 5, *b*_1_^II^ = 10 for ϑ^core^ = 0.0, and ϑ^core^ = 0.8.

### 5.2 Two-Component Mixture Analysis

**Figure 4:**
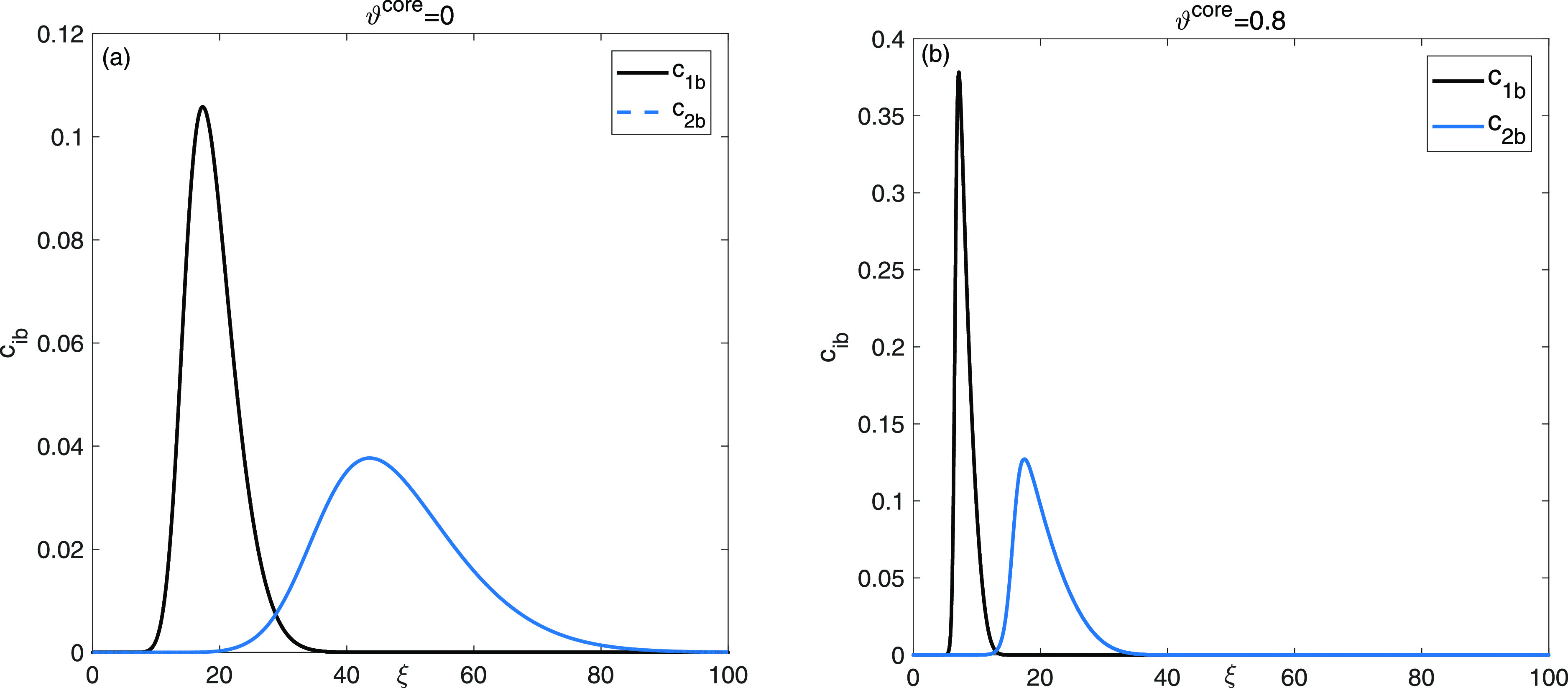
Two-component mixture analysis: comparison of elution curves for entirely porous (a): ϑ^core^ = 0.0 and cored beads with a small radius fraction (b): ϑ^core^ = 0.8.

In Figure 4, the impact of ϑ^core^ on the broadening of bands and duration of retention in the effluent breakthrough profiles is illustrated for a mixture of two-component. Elution curves for entirely porous particles exhibit substantial overlap, and the desired separation of both peaks along the baseline is not accomplished. However, when using core-particles with ϑ^core^ = 0.8, the elution peaks become sharper, the retention times of both constituents diminish, and the resolution of the mixture improves substantially. At ϑ^core^ = 0.8, it is evident that the two peaks are almost completely separated due to the reduction in bandwidth. Furthermore, the nondimensional time needed for complete elution of both peaks was reduced from 100 for fully porous beads with a core density of ϑ^core^ = 0 to 40 for core beads with a core density of ϑ^core^ = 0.8. This reduction in the elution time resulted in enhanced productivity in a competitive batch process.

**Figure 5:**
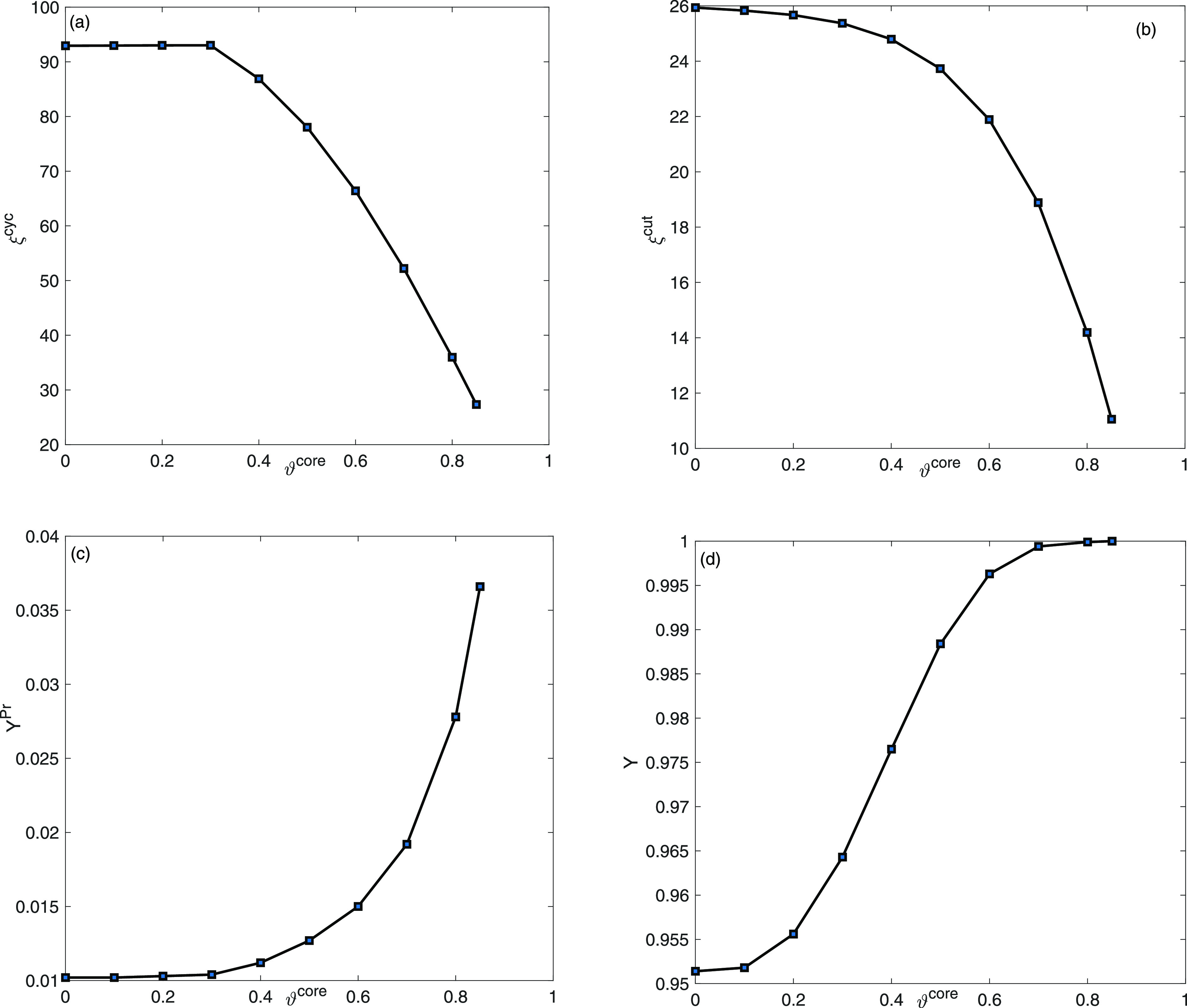
Two-component mixture analysis: plots of (a) ξ^cyc^, (b) ξ^cut^, (c) *Y*^Pr^, and (d) *Y* as a function of core radius fraction ϑ^core^.

To evaluate the process performance in the case of the two-component mixture, plots of ξ^cyc^, ξ^cut^, *Y*^Pr^, and *Y* are shown as described in (c.f.^36–39^) as a function of ϑ^core^ for 0 ≤ ϑ^core^ ≤ 0.85 are presented in Figure 5. It is evident that the cycle time decreases from 92.94 for entirely porous particles ϑ^core^ = 0 to 27.34 for core particles with a core density of ϑ^core^ = 0.85. In a similar manner, the cut time ξ^cut^ drops from 25.93 to 11.05. However, the productivity *Y*^Pr^ and yield *Y* continue to increase consistently with higher ϑ^core^ values as a consequence of enhanced adsorption activity in the presence of favorable sites for adsorption in the stationary bed, modeled by two independent adsorption sites.

**Figure 6:**
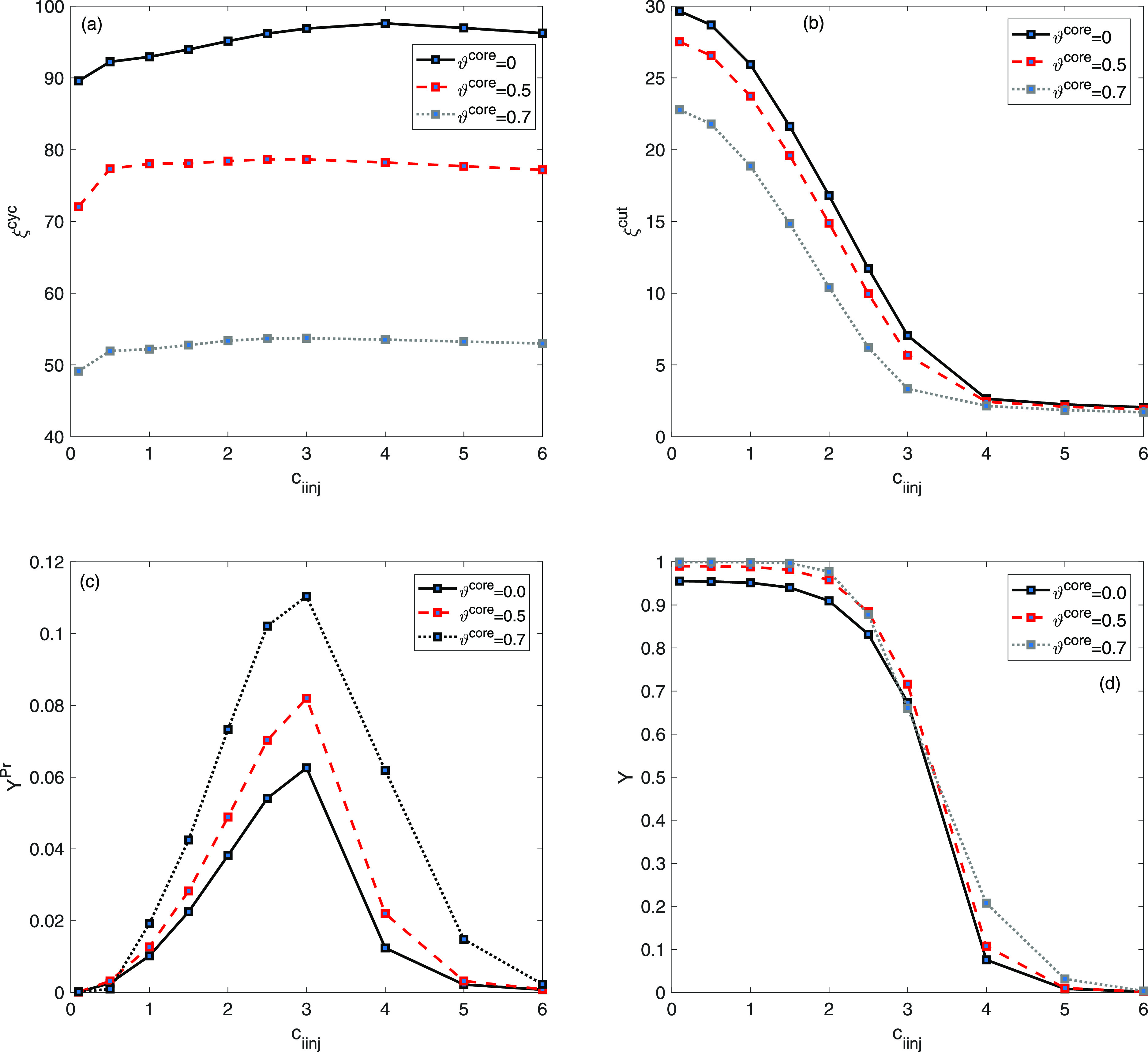
Two-component mixture analysis: plots of (a) ξ^cyc^, (b) ξ^cut^, (c) *Y*^Pr^, and (d) *Y* as a function of injected concentration *c*_iinj_.

Figure 6 displays the effects of nonlinear adsorption at higher injected concentration on the thickness of core radius fraction to predict the maximum level of productivity. For this particular case study, we have selected *c*_1inj_ = *c*_2inj_, and as the value of *c*_*i*inj_ increases, productivity experiences an initial increase and eventually reaches a maximum value for *c*_*i*inj_ = 3. Subsequently, the level of productivity experiences a decline and ultimately reaches a constant level for higher *c*_*i*inj_ values.

This fact could be evident from the results displayed in Figure 6. Table 2 enumerates the utmost values of productivity as well as other parameters at the specified values of ϑ^core^ and *c*_*i*inj_. It is depicted in Figure 6 and Table 2 that the maximum value of productivity had been obtained in the range of 1.0 ≤ *c*_*i*inj_ ≤ 3.0 for 0 ≤ ϑ^core^ ≤ 0.9. Within this range, the highest possible level of productivity can be observed when ϑ^core^ = 0.9 and *c*_*i*inj_ = 3. It is crucial to note that elevating the concentration of the feed corresponds to an increase in the extent of nonlinearity coefficients for sites I and II, respectively, as depicted from the isotherm formulation in eq 7. The significant decrease in the volume of the porous layer led to a notable decline in the loadability of the column.

**Table 2:** Optimal Values of the Process Performance Parameters

ϑ^core^	*c*_*i*inj_	*c*_1b_	*c*_2b_	*Y*^Pr^	*Y*	ξ^cyc^	ξ^cut^
0.0	3.0	2.9998	2.9999	0.0626	0.6734	96.8795	7.0515
0.5	3.0	2.9993	3.0000	0.0820	0.7162	78.6388	5.6852
0.7	3.0	2.9951	2.9999	0.1104	0.6603	53.735	3.3340
0.9	3.0	2.9873	2.9955	0.1172	0.2602	19.9001	1.7310

**Figure 7:**
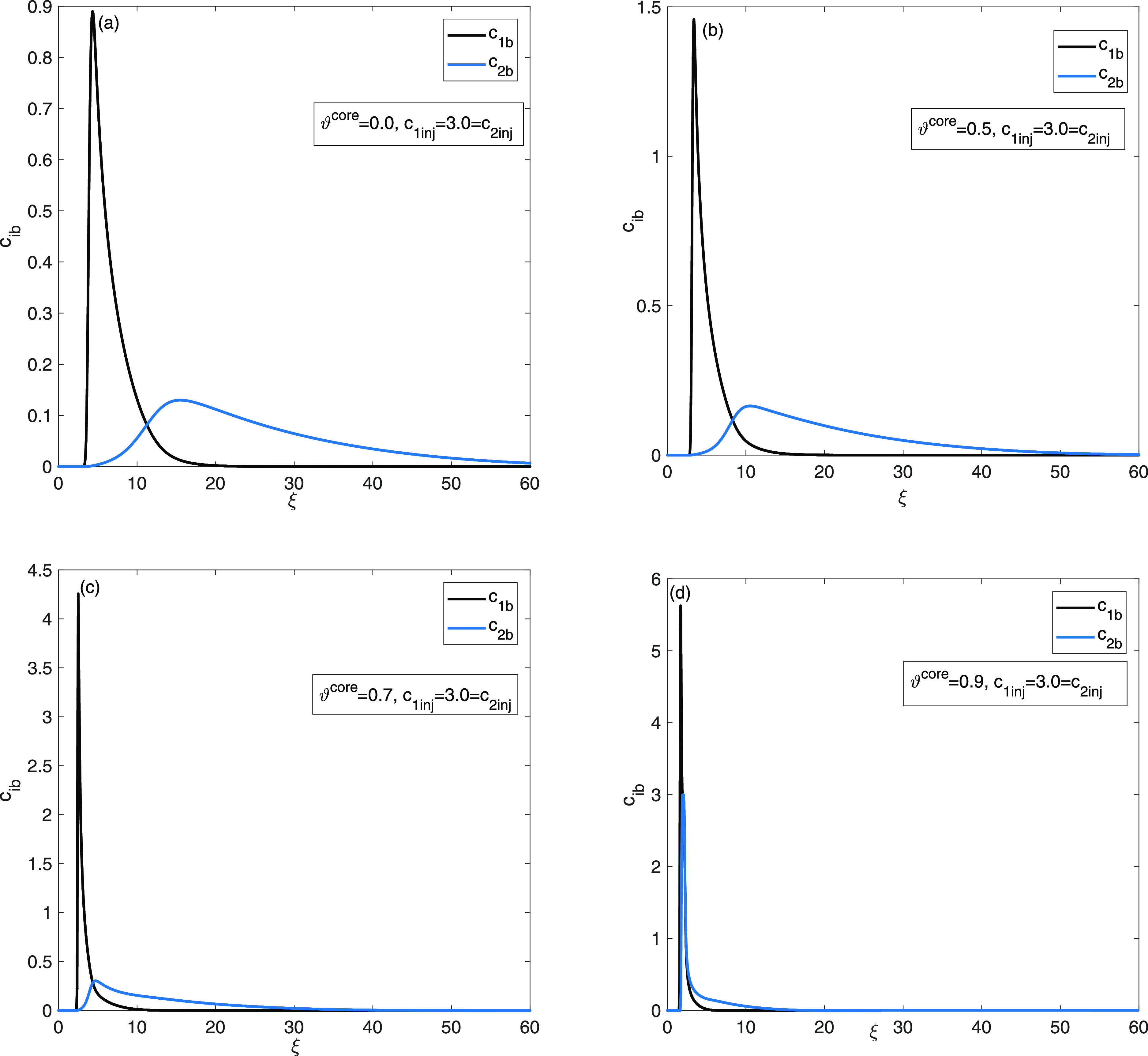
Two-component mixture analysis: comparison of elution curves for distinct ϑ^core^ values at specific *c*_*i*inj_ = 3 for *i* = 1, 2 to obtain maximum productivity level, (a) ϑ^core^ = 0, (b) ϑ^core^ = 0.5, (c) ϑ^core^ = 0.7, and (d) ϑ^core^ = 0.9.

**Figure 8:**
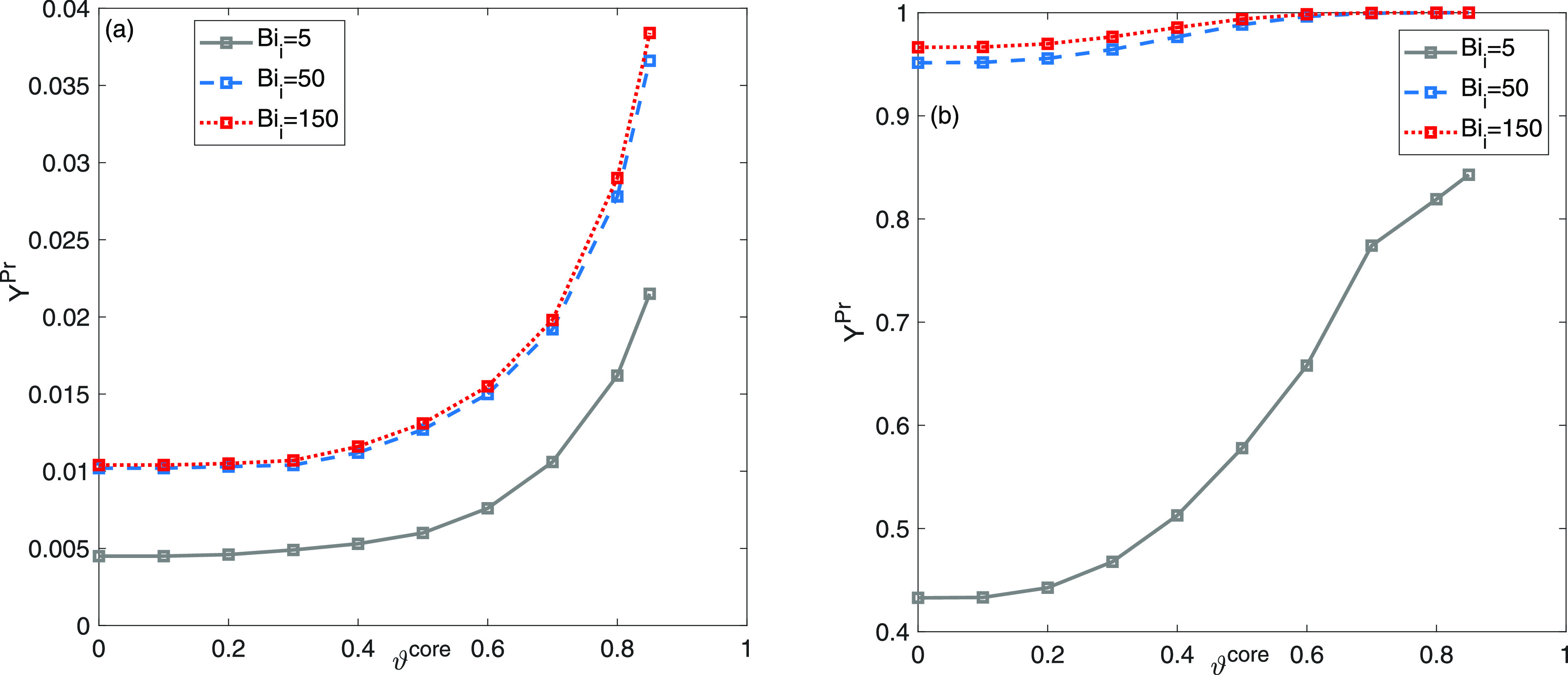
Two-component mixture analysis: impact of external mass-transfer resistance *Bi*_*i*_ on the plots of productivity (a) *Y*^Pr^ and yield (b) *Y* as a function of ϑ^core^.

Consequently, there was a decrease in both the productivity and the economy of the separation process. Plots in Figure 7 exhibits elution curves at the maximum productivity level, highlighting specific values of ϑ^core^ to investigate the influence of core thickness. Figure 8 evaluate the effects of the kinetic parameter *Bi*_*i*_. Plots in Figure 8 exhibit that optimum ϑ^core^ values for productivity devolve on the value of Biot number *Bi*_*i*_. Two different values of Biot numbers, such as *Bi*_*i*_ = 5 and *Bi*_*i*_ = 150 are taken into account when considering the result obtained in Figure 5c,d for *Bi*_*i*_ = 50 as a reference. Both a reduction in the rate of mass transport around the particles and an elevation in the rate of mass transport surrounding the particles yielded identical outcomes in terms of the ratio of the resistances to mass transfer at the exterior and interior. According to Figure 8a, it can be observed that the optimal values of core radius fraction, which result in the maximum productivity for capturing the initially eluting constituent, i.e, component one, shift toward more significant values as the Biot number decreases, indicating thinner shell layers. The variations in yield and productivity between Biot numbers of *Bi*_*i*_ = 50 and *Bi*_*i*_ = 150 are negligible. This indicates that the influence of transportation within the laminar boundary layer is almost insignificant when *Bi*_*i*_ = 50. Under such conditions, the configurations of the bands are predominantly determined by the resistances to internal mass transfer and longitudinal dispersion.

**Figure 9:**
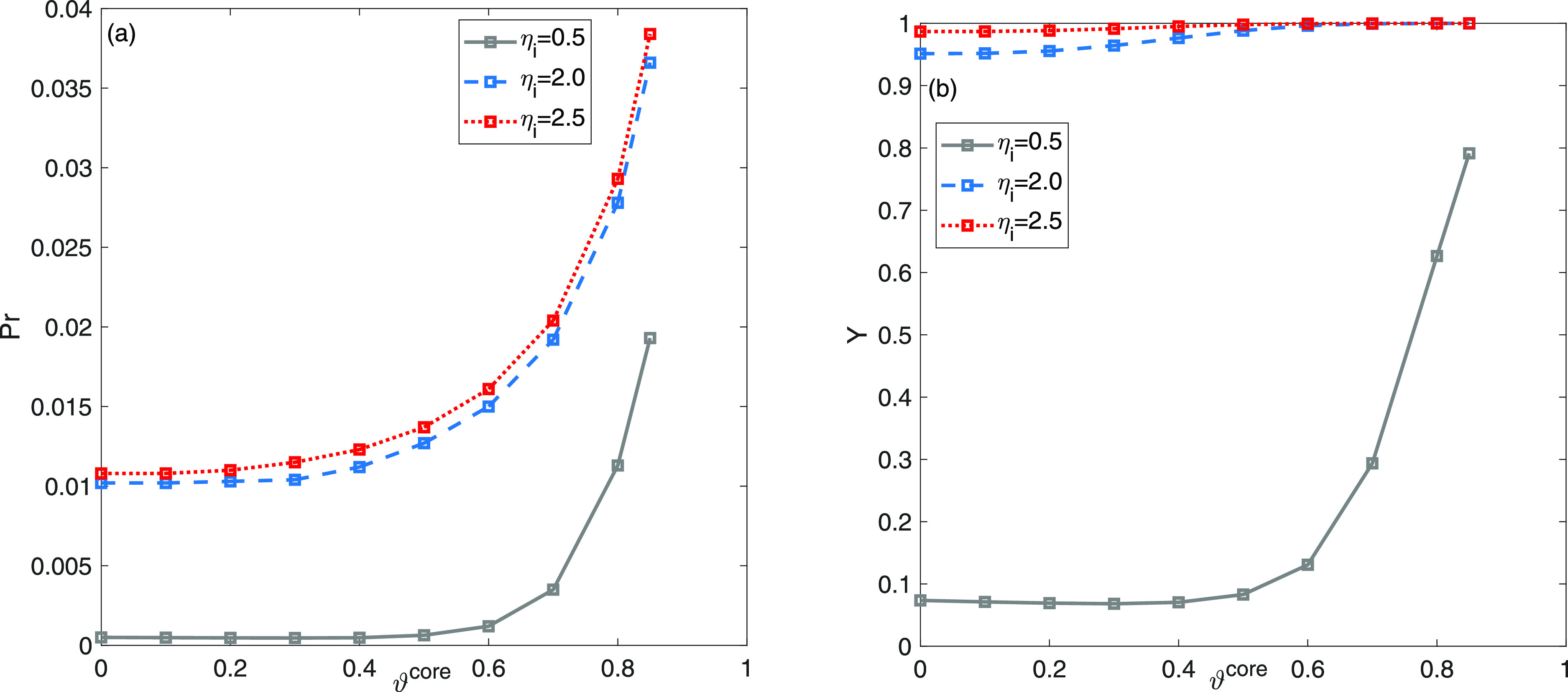
Two-component mixture analysis: impact of intraparticle diffusion resistance η_*i*_ on the plots of productivity (a) *Y*^Pr^ and yield (b) *Y* as a function of ϑ^core^.

The results portrayed in Figure 9 reveal the correlation between the core radius fraction values and the intraparticle diffusion parameter η_*i*_. Once again, two distinct values of the kinetic parameter η_*i*_, such as η_*i*_ = 0.5 and η_*i*_ = 2.5 are taken into account when considering the result obtained in Figure 5c,d for η_*i*_ = 2 as a reference. As depicted in Figure 9a, it is apparent that the optimal ϑ^core^ values, leading to maximum productivity for capturing the initial eluting component, exhibit a tendency to shift toward higher values, implying thinner core–shell layers with an increase in the parameter η_*i*_. Therefore, the presence of nonuniform adsorption sites and accelerated transfer rates within the shell prompt a reduction in the thickness of the shell layer. The recovery decreases concurrently with a reduction in intraparticle diffusion. It is important to highlight that these results, along with the findings presented in Figure 8, are specific to the provided composition of feed. Alterations in the optimal values would arise for different injected feed concentrations, as demonstrated in Figure 6.

### 5.3 Three-Component Mixture Analysis

**Figure 10:**
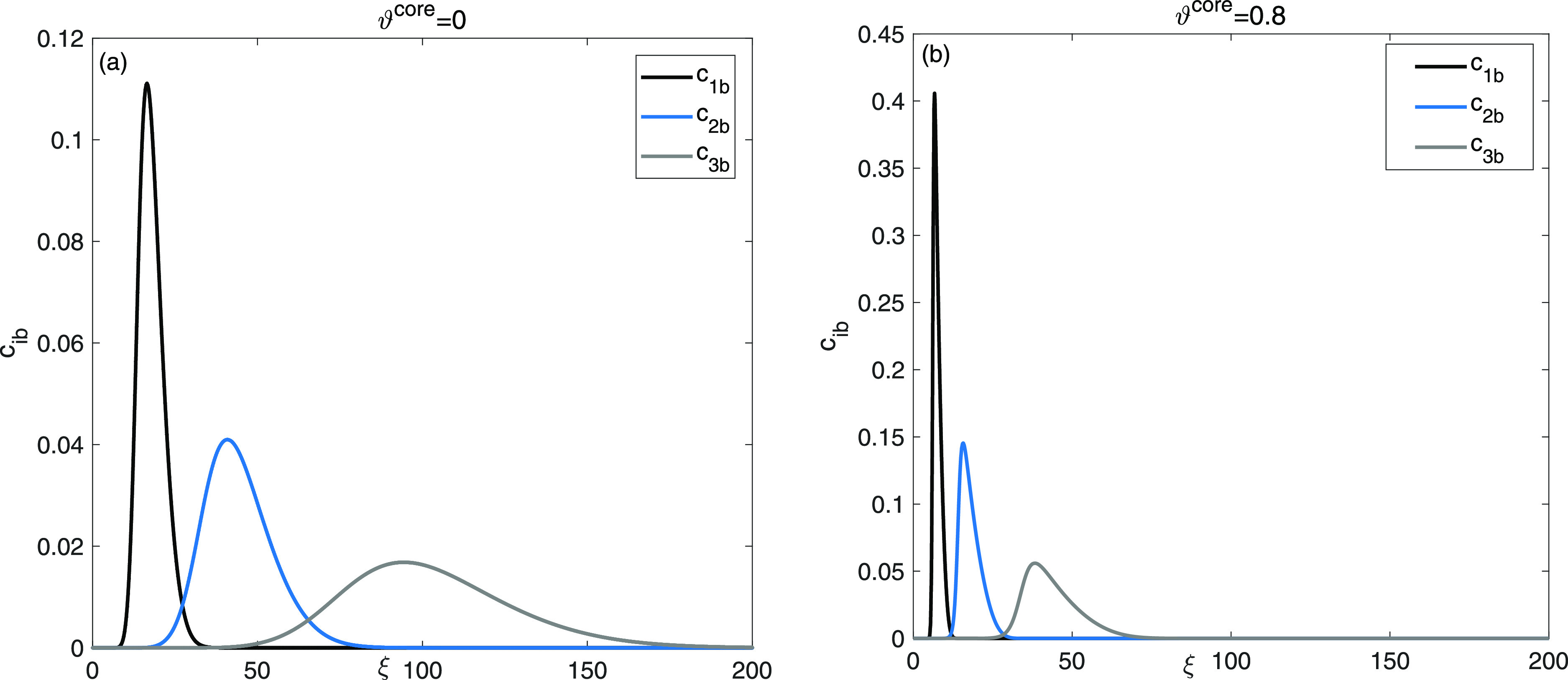
Multicomponent mixture analysis: comparison of elution curves for entirely porous (a) ϑ^core^ = 0.0 and cored beads with a small radius fraction (b) ϑ^core^ = 0.8.

To analyze multicomponent scenario, the influence of ϑ^core^ on band broadening and retention times of the elution curves are shown in Figure 10 for a three-component mixture. The complete list of model parameters utilized in the simulation studies is compiled in Table 1.

The characteristics of the elution profiles resemble those observed in the aforementioned case study of a two-component mixture. Notable effects observed for larger values of ϑ^core^ = 0.8 include the development of asymmetrical (effectively vertical) elution fronts, characteristic of the bi-Langmuir adsorption isotherm. Additionally, shorter retention times, enhanced separation for all mixture components, and increased peak heights are noticeable. A complete separation of the three peaks can be observed with a value of ϑ^core^ = 0.8. Moreover, the average dimensionless time needed for complete elution of the three peaks was reduced from 200 for fully porous particles ϑ^core^ = 0 to 95 for core particles with a core density of ϑ^core^ = 0.8.

## 6 Conclusions

The primary objective of this study was to execute a numerical simulation of the 1D, nonlinear GRM for liquid chromatography. A nonlinear GRM was developed to investigate the behavior of multicomponent solute flows in a single column adsorption process. The model incorporated a bi-Langmuir adsorption isotherm, considering independent adsorption occurring on two distinct adsorption sites. The primary emphasis of the study was examining the application of core–shell adsorbents in this context. The research extended and implemented a highly accurate, stable, and fast HR-FVS for the numerical solution of the model equations. The findings indicated that an increase in the core radius fraction resulted in reduced residence times and more pronounced peak shapes. Consequently, when core–shell adsorbents are utilized to pack a column with two independent adsorption sites, the separation efficiency of the column is expected to improve due to the decreased diffusion path within the adsorbents. Moreover, these particles provide analytical separations with modest injected volumes and concentrations. Performance specification criteria were assessed, taking into consideration the cycle times, through utilization of numerical simulations in order to determine the optimal values for the core radius fraction and injected feed concentration. Through the examination of specific parameter studies, it was discovered that the optimal thickness of the shell layer, which boosts the productivity of preparative chromatography, is dependent on various factors such as the injection concentration, the Biot number, and the intraparticle diffusion resistance. The model and numerical solutions presented are considered valuable for comprehending and optimizing processes, such as the purification of various compounds as well as for the development of appropriate core–shell particles.
